# Sodium-fluorescein-guided awake surgery for cerebral metastases located in eloquent brain areas: technical notes and preliminary experiences

**DOI:** 10.55730/1300-0144.5783

**Published:** 2023-11-11

**Authors:** Alper TÜRKKAN, Pınar OCAK, Marzieh KARIMI KHEZRI, Ahmet BEKAR

**Affiliations:** 1 Department of Neurosurgery, Medicana Hospital, Bursa, Turkiye; 2 Department of Neurosurgery, Faculty of Medicine, Uludağ University, Bursa, Turkiye

**Keywords:** Fluorescence-guided surgery, awake craniotomy, brain metastasis, sodium fluorescein

## Abstract

**Background/aim:**

Awake craniotomy (AC) maximizes the resection of lesions in eloquent brain areas while preserving functionality. Tumor delineation with intraoperative use of sodium fluorescein (NaFl) facilitates total resection. When used with AC, it may allow for safe resection without increasing the risk of postoperative neurologic deficits. This study investigated the efficacy and safety of the combined use of NaFl and AC for maximum safe resection in patients with brain metastases.

**Material and methods:**

Patients who underwent AC due to brain metastasis in the Department of Neurosurgery of Uludağ University’s Faculty of Medicine between January 1, 2018 and August 1, 2022, were retrospectively analyzed. The study comprised 2 patient groups: plain AC (pAC) and NaFl-guided AC (NaFlg–AC). Surgical outcomes related to fluorescence intensity, degree of resection, perioperative complications, and postoperative neurological factors were evaluated.

**Results:**

The pAC group included 16 patients (12 males, 4 females), and the NaFlg–AC group comprised 21 (13 males, 7 females). The mean patient ages for males and females were 61.4 years (61.4 ± 9.5 years) and 60.4 years (60.6 ± 12 years), respectively. The most common origin of the metastatic lesion was the lung in both the pAC and NaFlg–AC groups (n = 12 vs. n = 14, respectively). Gross total resection (GTR) was achieved in 85.7% of the patients in the NaFlg–AC group, whereas the GTR rate was 68.7% in the pAC group. There was no significant difference in GTR rates between the 2 groups (p = 0.254). The mean duration of the resection time was significantly shorter in the NaFlg–AC group (45.95 ± 7.00 min vs. 57.5 ± 12.51 min; p = 0.002). The patients’ Karnofsky Performance Status (KPS) score did not reach statistical significance at 6-month follow-up in either group compared to their preoperative baseline scores (p = 0.374). KPS did not show a significant difference between the 2 groups at any time.

**Conclusion:**

Fluorescence-guided resection in AC for metastatic tumors in sensory, motor, and cognitive areas is a feasible, safe, and convenient technique that significantly increases GTR rates and shortens operative time compared to conventional white light surgery without fluorescence guidance. It also does not increase the incidence of postoperative complications. With the combined use of AC and NaFl, ensuring clear and visible tumor margins during surgery and controlling patients’ neurological function in real-time are possible.

## 1. Introduction

Brain metastases are the most common malignancies observed in the central nervous system (CNS), and up to 20%–30% of cancer patients develop them at some point during the course of their disease [1–3]. Several types of malignant tumors can metastasize to the CNS. The most common primary tumors responsible for brain metastases are lung cancer, melanoma, renal cell carcinoma, breast cancer, and colorectal carcinoma [3–5].

Surgical total resection of brain metastases is crucial in improving patients’ quality of life and survival [5–7]. Therefore, surgery should be considered the first treatment option for such individuals. However, surgical resection of metastases located in functional brain regions or those near the eloquent areas is a challenge encountered during neurosurgery because, in addition to total resection, preservation of neurologic functions should be the primary goal during surgical planning [8,9],primary. Therefore, effective surgical methods and resection strategies to preserve neurological functions continue to evolve daily.

Awake craniotomy (AC) is a modern and advanced method in neurosurgery and plays an important role in the treatment of brain tumors. This surgical technique aims to preserve the functional regions of the brain and improve these functions during the removal of brain tumors [10]. There is increasing evidence that AC is an effective method for tumor removal in or near functional regions, resulting in a higher rate of resection, lower rates of neurological complications, and better overall survival [10–13].

During standard microsurgery, surgical interventions using white light provided by the operation microscope without special filters can sometimes be inadequate in assessing the extent of resection of cerebral metastatic masses. There may be difficulties in determining the site of cortical access and the margins of the mass, especially in deep mass lesions. Therefore, new techniques are necessary to enhance maximal and safe resection, and the use of fluorescein-guided surgery has recently gained attention and has been increasingly studied [14–18]. Fluorescein-guided surgery is based on visualizing the surgical field with special filters by administering fluorescent contrast material during the surgical procedure to obtain real-time visualization of the tumor.

Sodium fluorescein (NaFl) is a fluorescent dye currently used intraoperatively for brain tumor visualization, first used in 1948 by Moore et al. [19]. With the development of a special filter mode for microneurosurgical microscopes, the use of NaFl in the visualization of cerebral tumors has since evolved. NaFl accumulates in extracellular spaces in regions where the blood–brain barrier (BBB) is disrupted [18–24]. Therefore, in all tumor tissue that displays contrast enhancement on preoperative computer tomography (CT) and magnetic resonance imaging (MRI), NaFl accumulates according to the disruption of the BBB and facilitates visualization of the tumor intraoperatively through a special filter [18–24]. Thus, intraoperative use of NaFl makes tumor demarcation and total resection more feasible. Combined with AC, it may allow safe resection without increasing the risk of postoperative neurologic deficits.

The present study aims to investigate the efficacy and safety of using the NaFl and AC techniques together for maximized and safe surgical management in patients with brain metastases.

## 2. Method

After the study was approved by the local ethics committee, patients who underwent awake surgery at our center between 2018 and 2022 and pathology results that indicated “metastases” were retrospectively analyzed. All procedures performed in this study, which involved human participants, complied with the ethical standards of the institutional or national research committee and the 1964 Declaration of Helsinki and its subsequent amendments or comparable ethical standards. Informed consent was obtained from all participants.

The data obtained from the patients who underwent AC before the introduction of the special filter microscope at our facility (2018–2020), and thus without the intraoperative use of NaFl (plain AC group, (pAC)), were compared to those who underwent NaFl-guided AC (2020–2022) (NaFlg–AC group) in terms of the extent of resection, postoperative complications, length of hospital stay, and postoperative Karnofsky Performance Status score (KPS) [25] (Table 1).

### 2.1. Criteria for awake surgery

Patients with lesions located next to functional brain structures, patients unable to cooperate during the awake procedure, or those with a motor weakness greater than 3 points on a 5-point Lovett scale were considered unsuitable for awake surgery. Relative contraindications for AC were severe asthma, chronic obstructive pulmonary disease, panic disorder, and depression.

### 2.2. Surgical procedure

All patients underwent AC under sedoanalgesia. Vital parameters (mean arterial blood pressure, heart rate, oxygen saturation, and body temperature) and neurological examinations were monitored throughout the procedure. Furthermore, perioperative alterations in neurological status were reported and recorded instantaneously.

All patients underwent a craniotomy large enough to achieve maximum safe resection. In the pAC group, the standard microsurgical procedure was followed for tumor resection, whereas predefined NaFl was administered intravenously at a dose of 5 mg/kg after anesthesia induction in the NaFlg–AC group. A NaFl-specific filter-assisted neurosurgical operative microscope was used to visualize the tumor during resection (Leica OH6, equipped with a FL560 filter, Leica Microsystems, Wetzlar, Germany), which was mainly based on the identification of stained tumor tissue. Perioperatively, the surgical resection cavity was visualized under a bright-colored field and fluorescence to assess the degree of resection. Moreover, the image of the tumor under a special filter microscope was evaluated according to the degree of fluorescent staining: (“yellow–green color/useful” and “no staining/not useful”). Maximum safe resection was defined as the maximum possible tumor resection without causing new neurological deficits.

In both groups, resection was halted when new neurologic deficits such as speech arrest or motor weakness were detected. Intraoperative seizures were managed with ice-cold lactated Ringer’s solution. After tumor resection, mild sedation with remifentanil, propofol, and pain control medications were used during wound closure.

All patients were followed up postoperatively in the intensive care unit for at least 24 h. New neurological deficits were defined as “transient” (if diminished within 30 days after surgery) or “permanent” (if present after a 30-day follow-up).

All patients were screened with preoperative 1-mm gadolinium-enhanced brain MRI, early postoperative (first 24 h) cranial CT, and control MRI. All neuroimaging samples were reviewed by an independent neuroradiologist and the authors of this study for residual contrast-enhancing tumor tissue. The resection was considered gross total resection (GTR) when no obvious visible tumor at the time of surgery or on postoperative MRI was observed. The resection was considered a subtotal resection when the residual tumor was greater than 5% of the total tumor volume. Surgical outcomes related to fluorescence intensity, degree of resection, perioperative complications, and KPS scores were evaluated comparatively.

### 2.3. Statistical analysis

Continuous variables were expressed in terms of mean ± standard deviation. Moreover, according to normality test results, an independent samples t-test was used to compare the 2 groups. Categorical variables were presented as frequency and percentage values (n; %) and compared using Pearson’s chi-square test. Statistical analysis was performed using GraphPad Prism 7 (GraphPad Software, San Diego, CA, USA). A p-value of <0.05 indicated statistical significance.

## 3. Results

A total of 37 patients meeting our inclusion criteria underwent tumor resection with AC. The pAC group included 16 patients (12 males, 4 females) who were operated on before introducing the special NaFl-filtered microscope at our facility. The NaFlg–AC group comprised 21 patients (13 males, 8 females). Female and male patients were equally distributed between groups (p = 0.4912). The mean ages of the male and femalepatients were 61.4 ± 9.5 years (range: 43–77 years) and 60.6 ± 12 years (range: 30–76 years) in the pAC and NaFlg–AC groups, respectively. There was no significant difference between groups in terms of age (p = 0.822). Preoperative KPS was ≥70 in all patients. Specifically, the KPS was 85 ± 10 in the pAC group and 83.8 ± 10 in the NaFlg–AC group. Headache was the most common presenting symptom (68.7 vs. 85.7%), followed by weakness in one or more extremities (62.5% vs. 71.4%) in the pAC and NaFlg–AC groups. Eleven cases (68.8%) in the pAC group and 9 (42.9%) cases in the NaFlg–AC group had right-sided lesions. The lesions were mostly located in the motor/premotor areas in the pAC and NaFlg–AC groups (87.5% vs. 76.2%, respectively).

In 20 (95.2%) patients in the NaFlg–AC group, yellow–green fluorescent staining was observed, which markedly increased tumor visibility under the microscope’s fluorescence mode (Figure). This staining was considered “useful.” Inadequate NaFl staining was observed in one case (4.8%) (Table 2).

The intraoperative period was uneventful in 12 (75%) and 15 (71.4%) patients in the pAC and NaFlg–AC groups, respectively. The most common intraoperative complication was seizure, which occurred in 2 patients per group (12.5% vs. 9.5%, respectively). Seizures were controlled using irrigation with an ice-cold solution and administrating antiepileptic medication; subsequently, the surgical procedure was resumed. There was no significant difference between groups in terms of intraoperative complications (p = 1) (Table 3).

The mean duration of the resection during surgery was significantly shorter in the NaFlg–AC group than in the pAC group (45.95 ± 7 min vs. 57.5 ± 12.51 min; p = 0.002).

GTR was achieved in 85.7% (18/21) of the NaFlg–AC group patients. Conversely, the GTR rate was 68.7% (11/16) in the pAC group. Although there was a tendency for higher GTR rates using NaFl, this difference failed to reach statistical significance (p = 0.254).

The most common origin of a metastatic lesion was the lung in both the pAC and NaFlg–AC groups (n = 12; 75% vs. n = 14; 66.6%, respectively). This was followed by the breast (n = 2; 12.5%), gastrointestinal tract (n = 1; 6.7%), and malignant melanoma (n = 1; 6.7%) in the pAC group. In the NaFlg–AC group, metastasis originating in the breast (n = 3; 14.2%), kidney (n = 2; 9.5%), gastrointestinal tract (n = 1; 4.7%), and testis (n = 1; 4.7%) was detected in decreasing order of frequency after metastasis from lung cancer.

Postoperative neurological examination revealed deterioration compared to presurgical neurological status in 2 (12.5%) of the patients in the pAC group. At 1-month follow-up, neurological examination of one of these 2 patients was improved and restored to the state at the time of preoperative neurologic examination. Therefore, permanent new-onset neurologic deficit was evident in 1 patient (6.3%) in the pAC group. Furthermore, postoperative neurologic examination revealed deterioration in 4 (19%) patients in the NaFlg–AC group (%19). At 1-month follow-up, neurological examination of 3 of these 4 patients was improved and restored to the state at the time of preoperative neurologic examination. Therefore, permanent new-onset neurologic deficit was evident in 1 patient (4.8%) in the NaFlg–AC group. There was no statistical significance between groups regarding new-onset neurological deficits at 1-month and 6-month follow-ups (p = 1) (Table 4).

The mean length of the hospital stay was 3.5 ± 0.5 days for the patients in the pAC group, whereas those in the NaFlg–AC group were discharged after a mean stay of 3.28 ± 0.45 days. The difference did not reach statistical significance (p = 0.176).

Statistical analysis showed no significant difference between the mean value of the patients’ KPS scores in the pAC and NaFlg–AC groups, both upon admission (85 ± 10 vs. 83.8 ± 10; p = 0.72) and at discharge (84.3 ± 13.2 vs. 83.3 ± 13; p = 826). At the 6-month follow-up, the mean KPS values were 90 ± 11.2 and 89 ± 10.8 for the pAC and NaFlg–AC groups, respectively. The patients’ KPS scores did not reach statistical significance at the 6-month follow-up in either group compared to their preoperative baseline scores (p = 0.374). KPS did not show a significant difference between the 2 groups at any time.

No NaFl-related side effects or complications were noted during the postoperative course. There was no surgery-related mortality in the present series.

## 4. Discussion

In the present study, we report a series of 37 patients with intracranial metastases who were successfully operated on with AC under local anesthesia and sedation. Low-dose NaFl was administered to 21 of these patients (NaFlg–AC group); it accumulated in the tumor tissue and provided intraoperative fluorescence. Using the special filter in the surgical microscope, a yellow–green color change was visualized, allowing us to distinguish the margins of the tumor tissue clearly in 95.2% of the cases. Thus, normal tissue manipulation was minimized, and microsurgical dissection from the surrounding tissue was achieved. The color change was visible for identification of the cortical incision site, which made the surgical method more reliable. In the subcortically located masses, the surrounding edematous tissue was observed to have a brighter yellow color than the normal tissue, which was also less green than the tumor tissue (Figure). Therefore, the NaFl application visualized the location of the surgical site intraoperatively, and since AC was performed, we were able to monitor the patients’ neurologic functions. Consequently, NaFl facilitated the total removal of metastatic tumors. The total resection rate was 85.7% in the fluorescence-applied group. In contrast, we achieved GTR in a much lower percentage of patients who underwent pAC (68.7%). However, this difference failed to reach statistical significance, probably due to the low number of cases in the present cohort.

For metastatic brain lesions requiring multimodal treatment with chemo- and/or radiotherapy, a rapid transition from surgery to the initiation of clinical therapies is crucial. Studies have shown that AC is associated with reduced length of stay in the intensive care unit and shorter hospital stays than other neurosurgical procedures performed under general anesthesia [7,10,26]. Previous reports have also demonstrated that AC is associated with significantly higher GTR rates and lower rates of permanent neurological deficit [7]. In a series of 76 patients undergoing maximum safe resection for primary and metastatic brain tumors, Groshev et al. reported that AC was associated with a shorter hospital stay and lower rates of postoperative complications [7]. Furthermore, in a study in which Chua et al. reviewed 7 published studies of AC performed for brain metastases, the authors reported a GTR rate of 61%, a supramarginal resection rate of 32%, and a subtotal resection rate of 7% in the patients. They reported improvement in the neurological function of patients undergoing AC, indicating that it should be considered a useful technique to optimize outcomes in brain metastases in significant regions [10].

Our study observed that patients who underwent AC tended to tolerate the transition to subsequent treatment well since they experienced shorter stays in the intensive care unit and hospital. Approximately 75% of the patients in our series were discharged within the first 48 h after surgery. Our study achieved GTR in 83% of the patients, which is consistent with the literature. However, the amount of resection in metastatic tumors is limited to the regions of motor and language functions. To ensure that patients continue to have a high quality of life, preservation of functions should be prioritized over radical tumor removal. Although it is possible to define functional regions intraoperatively with AC, determining the tumor resection margins remains challenging. Even if assistive devices, such as ultrasound or neuronavigation, are used intraoperatively in AC, for most neurosurgeons, the ability to distinguish the margins between normal tissue and tumor tissue during tumor resection is of utmost importance [20]. In the current study, we were able to visualize the tumor tissues intraoperatively using NaFl in the NaFlg–AC group patients.

NaFl is one of the most commonly used reagents for intraoperative visualization during tumor removal [27]. Unlike other fluorescent substances, it does not accumulate intracellularly in tumor cells. Moreover, it is distributed and accumulates in the extracellular space along regions of the brain where the blood–brain barrier is disrupted [28]. Therefore, it accumulates in all tumors, demonstrating contrast uptake on preoperative CT and MRI [28]. NaFl can be administered intravenously, so its dose adjustment and administration are easier and cheaper than other fluorescent agents. It has a low side effect profile [19,22,23], and previous studies have supported well-staining in cerebral metastases with NaFl, which generally displays intensive enhancement on cranial CT and MRIs. For example, after NaFl-guided surgery in 30 patients with cerebral metastases, Schbesh et al. reported satisfactory staining in 90% of the patients. GTR was achieved in 83.3% of the cases in the same study [21]. Reporting the results of NaFl-guided surgery in 95 patients with cerebral metastases from different primary cancers, Höhne et al. found bright fluorescent staining in 95% of the patients compared to a rate of GTR in 83% of the cases ([24]. In a study of 38 patients who underwent either NaFl-guided or white light surgery, Xiao et al. found significantly higher GTR rates in the NaFl-guided surgery group [22]. Similarly, Kofoed et al. compared NaFl-guided surgery and white light surgery in 117 patients with cerebral metastases, reporting a statistically higher degree of resection (94% vs. 84%) in the NaFl-guided surgeries [23].

Based on our experience, we hypothesize that visualizing the tumor may potentially provoke neurosurgeons’ tendency to perform more aggressive resection. Specifically, during a tumor resection surgery under white light and in cases with lesions located near functional brain regions, the surgeon would try to perform a much more controlled resection to avoid causing new-onset neurologic deficits. However, obvious visualization of the tumor tissue, such as the use of intraoperative NaFl, may encourage the surgeon to attempt further resection. Notably, NaFl is not a tumor-specific fluorescent dye [28,29]. Thus, it can accumulate in any areas where the BBB is disrupted [28,29], including iatrogenically injured areas of the adjacent brain tissue. It has been determined that edematous brain tissue around the tumor acquires a brighter yellow color than normal brain tissue but not as much as the tumor tissue itself [7,29]. Therefore, attempting to remove all NaFl-stained areas may increase the likelihood of damage to functional regions, leading to new neurological deficits. Hence, based on the surgeon’s experience, it is important to work intermittently under both standard white light and fluorescent illumination to assess the degree of resection. In the present study, the total resection rate was higher in the NaFlg–AC group. Notably, the number of patients who developed new neurologic deficits in the early postoperative period was also slightly higher than that of the pAC group (19% vs. 12.5%). In patients with progressive intraoperative neurological deficit, resection was stopped immediately to preserve the functional status, regardless of the extent of resection. Therefore, although the KPS scores were lower than the preoperative status in these patients, reestablishing their initial neurological status in the postoperative months was possible. At the 6-month follow-up, there was no difference between the 2 groups in terms of their KPS scores.

Patient compliance with the surgical procedures in patients undergoing AC is closely related to the duration of the operation [11,30]. Notably, the ability to perform an effective surgical procedure in a shorter time has been shown to facilitate proper cooperation of patients during awake surgeries while closely tracking neurological examination. We noted shorter operative times in the NaFlg–AC group compared to the standard microsurgery group, which we believe was the result of faster and safer surgery due to successful visualization of the tumor localization, as well as the resection margins in patients who underwent NaFl-guided surgery.

To our knowledge, we are the first to present data on the efficacy of using NaFl in combination with AC in patients with intracranial metastases located near functional brain regions. We did not observe any local or systemic side effects related to using this fluorescent agent, including allergic side effects. The most common intraoperative complication in both groups was seizure in the present series, which was not attributed to intraoperative use of NaFl as intraoperative seizure is already a well-documented complication of AC, reported to occur in between 5%–16% of cases in previous studies [31,32]. Seizure activity in the course of awake surgery can be triggered during the cortical incision and resection of the lesion and can potentially jeopardize the operation’s success of the operation [31–33]. Despite premedication with antiepileptic loads prior to surgery, we observed intraoperative seizure in 4 (10.8%) of our cases, which were controlled by irrigation of the surgical field using ice-cold lactated Ringer’s solution and administration of intravenous propofol. After controlling the seizure, the AC procedure was safely initiated in all patients.

Based on the data presented above, we conclude that the real-time information obtained during surgery is an undisputed advantage of NaFl-guided surgery. AC provides instantaneous control of the patient’s neurological function, and NaFl provides clear and visible tumor margins. Thus, the combined use of both methods has a synergistic effect. We consider this an association that complements the scope of safe resection and increases patient safety.

The retrospective nature of this study, which included a small number of patients, is a limitation. Our results need to be confirmed by randomized controlled trials with larger sample sizes.

## 5. Conclusion

NaFl-guided surgery facilitates tumor demarcation and total resection. Combined with AC, it may allow for safer resection without increasing the risk of postoperative neurologic deficits after surgery.

## Figures and Tables

**Figure and Figure Legend:**
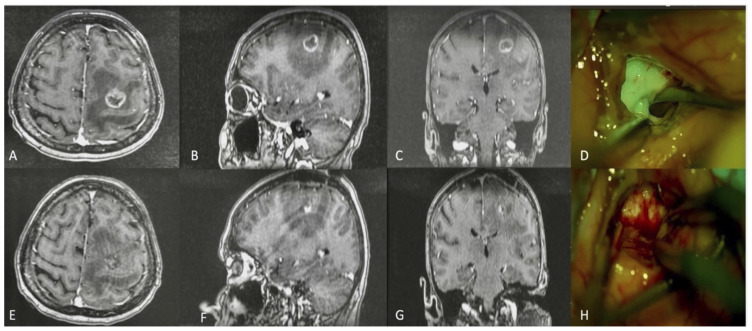
Intraoperative tumor visualization. Preoperative axial (A), sagittal (B), and coronal (C) gadolinium-enhanced MRI images demonstrating a tumoral lesion in the left premotor area in a 42-year-old male; (D): intraoperative real-time visualization of the tumor under the YELLOW 560-nm filter; (E–G): postoperative MRI confirmed total tumor resection; (H): cavity after tumor resection.

**Table 1:** Karnofsky performance status.

%	Specific criteria
100	Normal; no complaints; no evidence of disease
90	Able to carry on normal activity; minor signs or symptoms of disease
80	Normal activity is done with effort; some signs or symptoms of disease
70	Cares for self; unable to carry on normal activity or do active work
60	Requires occasional assistance but is able to care for most personal needs
50	Requires considerable assistance and frequent medical care
40	Disabled; requires special care and assistance
30	Severely disabled; hospital admission indicated, although death not imminent
20	Very sick; hospital necessary; active supportive treatment required
10	Moribund

**Table 2:** Demographical and clinical characteristics of patients included in the study.

	pAC group n (%)	NaFlg–AC group n (%)	*p-value*
**Number of patients**	16 (43.2)	21 (56.8)	
**Sex**			
Male	12 (75)	13 (61.9)	*0.4912*
Female	4 (25)	8 (38.1)	
**Ages, years ± SD**	61.4 ± 9.5	60.6 ± 12	*0.822*
**Side**			
Right	11 (68.8)	9 (42.9)	*0.1845*
Left	5 (31.2)	12 (57.1)	
**Localization**			
Motor/premotor area	14 (87.5)	16 (76.2)	*0.6745*
Speech areas	2 (12.5)	4 (19)	
Visual cortex	-	1 (4.8)	
**Signs and symptoms**			
Headache	11 (68.7)	18 (85.7)	
Weakness	10 (62.5)	15 (71.4)	
Sensorial deficit	10 (62.5)	12 (57.1)	
Seizure	9 (56.2)	12 (57.1)	
Cognitive deficit	4 (25)	6 (28.5)	
Speech deficit	2 (12.5)	3 (14.2)	
**Extent of surgery** *			
GTR	11 (68.7)	18 (85.7)	*0.254*
STR	5 (31.3)	3 (14.3)	
**Pathology**			
Lung	12 (75)	14 (66.6)	
Breast	2 (12.5)	3 (14.2)	
Malignant melanoma	1 (6.7)	-	
Kidney	-	2 (9.5)	
Testis	-	1 (4.7)	
Gastrointestinal tract	1 (6.7)	1 (4.7)	
**Mean resection time, minutes ± SD**	57.5 ± 12.11	45.95 ± 6.83	***0.002*** *
**Length of hospital stay, days ± SD**	3.5 ± 0.5	3.28 ± 0.45	*0.176*
**KPS, mean ± SD**			
Preoperative	85 ± 10	83.8 ± 10	*0.72*
At discharge	84.27 ± 13.21	83.3 ± 13.2	*0.826*
6-month follow-up	90 ± 11.2	86.7 ± 10.8	*0.374*

**GTR:** gross total resection; **KPS**: Karnofsky performance score, **STR:** subtotal resection.

* Statistically significant

**Table 3:** Distribution of intraoperative complications during AC.

Intraoperative complications	pAC group n (%)	NaFlg–AC group n (%)
Seizure	2 (12.5)	2 (9.5)
New speech deficit	-	1 (6.2)
New motor deficit	1 (6.2)	2 (9.5)
Nausea/vomiting	1 (6.2)	1 (4.7)

**Table 4:** Postoperative neurological status of patients who underwent AC.

Postoperative neurological status	pAC group n (%)			NaFlg–AC group n (%)		
	No change	Improved	Worsened	No change	Improved	Worsened
**Early postoperative period**	8 (50)	6 (37.5)	2 (12.5)	12 (57.1)	5 (23.8)	4 (19)
**1-month follow-up**	9 (56.2)	6 (37.5)	1 (6.3)	15 (71.4)	5 (23.8)	1 (4.8)
**6-month follow-up**	9 (56.2)	6 (37.5)	1 (6.3)	15 (71.4)	5 (23.8)	1 (4.8)
